# Supplementary material for: Efficacy of electronic travel aids for the blind and visually impaired during wayfinding
Source: Sci Rep. 2026 Jan 28;16:6423. doi: 10.1038/s41598-026-37578-9 (PMC12909938; doi:10.1038/s41598-026-37578-9)
Supplement: Supplementary file 1 — Supplementary Material 1 [file 41598_2026_37578_MOESM1_ESM.docx]

Supplementary Materials for

Efficacy of Electronic Travel Aids for the Blind and Visually Impaired During Wayfinding

Claire E. Pittet *et al.*

Corresponding authors: Claire E. Pittet, claire.pittet@chuv.ch and Micah M. Murray, micah.murray@chuv.ch

**The PDF file includes:**

Tables S1 to S5

**Table S1.** Overview of ETA devices with their original and archived Internet Archive URLs. Archived links ensure continued accessibility if original websites become unavailable. The BuzzClip and Sunu Band were discontinued at the time of the study.

| Device | Original URL | Archived URL |
| --- | --- | --- |
| WeWalk | [https://www.wewalk.io](https://www.wewalk.io/) | <https://web.archive.org/web/20251016071614/https://wewalk.io/en> |
| Ultracane | [https://www.ultracane.com](https://www.ultracane.com/) | <https://web.archive.org/web/20250811082632/https://ultracane.com> |
| GoSense | [https://www.gosense.com](https://www.gosense.com/) | <https://web.archive.org/web/20251016072121/https://www.gosense.com> |
| BuzzClip | No longer available | [https://web.archive.org/web/20221206093752/https://imerciv.com](https://web.archive.org/web/20221206093752/https://imerciv.com/) |
| MiniGuide | [https://www.gdp-research.com.au](https://www.gdp-research.com.au/) | <https://web.archive.org/web/20251016072836/https://www.gdp-research.com.au> |
| Sunu Band | No longer available | [https://web.archive.org/web/20211208194032/https://www.sunu.com](https://web.archive.org/web/20211208194032/https://www.sunu.com/) |
| NOA | [https://www.biped.ai](https://www.biped.ai/) | <https://web.archive.org/web/20251016073203/https://www.biped.ai> |
| Glide | [https://glidance.io](https://glidance.io/) | <https://web.archive.org/web/20251016073515/https://glidance.io> |
| .lumen | [https://www.dotlumen.com](https://www.dotlumen.com/) | <https://web.archive.org/web/20251016073842/https://www.dotlumen.com> |

**Table S2.** Performance metric differences between outward and return trials in the obstacle detection task. Wilcoxon signed-rank tests were performed as data was not normal. *W* values are reported. Values are presented as mean ± standard deviation (M ± SD). PPWS is expressed in percentage (%). All other values are event occurrences except heart rate, measured in beats per minute. Effect sizes are rank biserial correlation *r_rb_.* *P*-values are reported both uncorrected (*p_uncorr_*) and corrected for multiple comparisons using the Benjamini–Hochberg procedure (*p_corr_*).

| **Metric** | **Outward** | **Return** | **Statistic** | ***p*_uncorr_** | ***p*_corr_** | **Effect size** |
| --- | --- | --- | --- | --- | --- | --- |
| PPWS (%) | 0.58 ± 0.16 | 0.65 ± 0.17 | *W* = 35.00 | **<0.001***** | **<0.001***** | -0.910 |
| Cane contact | 1.85 ± 1.39 | 1.64 ± 1.06 | *W* = 82.50 | 0.400 | 0.466 | 0.214 |
| Body contact | 1.44 ± 1.21 | 1.26 ± 1.27 | *W* = 106.50 | 0.321 | 0.466 | 0.228 |
| Exploration | 0.15 ± 0.49 | 0.21 ± 0.47 | *W* = 10.50 | 0.588 | 0.588 | -0.250 |
| Gait change | 1.46 ± 1.35 | 1.59 ± 1.41 | *W* = 132.00 | 0.397 | 0.466 | -0.188 |
| Deviation | 0.10 ± 0.38 | 0.00 ± 0.00 | *W* = 0.00 | 0.174 | 0.405 | 1.000 |
| Heart rate (BPM) | 83.35 ± 10.40 | 82.88 ± 18.50 | *W* = 241.00 | 0.152 | 0.405 | -0.276 |

**Table S3.** Responses to ternary-choice questions in the obstacle detection task. McNemar's test was used to assess differences between conditions. Percentages reflect the proportion of participants who answered "yes" for each question under each condition. Δ% Yes indicates the difference in "yes" responses between NOA and BuzzClip. *P*-values are reported both uncorrected (*p_uncorr_*) and corrected for multiple comparisons using the Benjamini–Hochberg procedure (*p_corr_*).

| **Question** | **%Yes NOA** | **%Yes BuzzClip** | **Δ% Yes** | ***p*_uncorr_** | ***p*_corr_** |
| --- | --- | --- | --- | --- | --- |
| Complement to cane | 100 | 53.85 | 46.15 | **0.031*** | **0.047*** |
| Increased mobility on familiar routes | 61.54 | 38.46 | 23.08 | 0.375 | 0.375 |
| Increased mobility on unfamiliar routes | 76.92 | 23.08 | 53.85 | **0.016*** | **0.047*** |

**Table S4.** Summary of participants’ responses to open-ended questions regarding learning difficulties, advantages, and disadvantages of NOA and the BuzzClip. Numbers in parentheses indicate the number of participants mentioning each aspect.

| Question | NOA | BuzzClip |
| --- | --- | --- |
| Difficult learning aspects | None (6), difficulty with sound spatialization (4), difficulty with device setting (3) | None (7), weak vibrations (4), imprecision (2) |
| Mobility advantages | Precision (8), easy interpretation (4), AI functionalities (4), flexibility / all-in-one nature (3), added safety and independence (3) | Lightweight / size (7), simplicity (3), potential utility for deaf-blind and children (1) |
| Mobility disadvantages | Weight / size (8), difficult interpretation of cues (4), none (2) | Weak vibrations (6), frequent vibrations (5), imprecision (5), none (1) |

**Table S5.** Data on participants demographics collected during the initial questionnaire.

| **Demographic** | **Summary** |
| --- | --- |
| Age (years) | mean = 55.8 (SD = 19.8), range: 25 – 85 |
| Sex | 8 M (61.5%), 5 F (38.5%) |
| Vision level | 5 low vision (38.5%), 4 total (30.8%), 4 severe (30.8%) |
| Congenital | 9 no (69.2%), 4 yes (30.8%) |
| Employment status | 5 retired (38.5%), 4 employed (30.8%), 4 unemployed (30.8%) |
| Living situation | 8 family (61.5%), 5 alone (38.5%) |
| Living location | 8 urban (61.5%), 5 rural (38.5%) |
| Frequency on familiar routes | 7 daily (53.8%), 3 several times a week (23.1%), 2 weekly (15.4%), 1 rarely (7.7%) |
| Frequency on unfamiliar routes | 5 rarely (38.5%), 4 never (30.8%), 3 several times a week (23.1%), 1 weekly (7.7%) |
